# Integrative RNA-seq and iRIP-seq analysis links SNRPA overexpression to transcriptomic and splicing alterations in hepatocellular carcinoma cells

**DOI:** 10.3389/fonc.2026.1800728

**Published:** 2026-05-01

**Authors:** Qingyao Chang, Yidi Wang, Jun Xu

**Affiliations:** 1 First Clinical College, Shanxi Medical University, Taiyuan, China; 2 School of Management, Shanxi Medical University, Taiyuan, China; 3 Department of Hepatobiliary Surgery, First Hospital of Shanxi Medical University, Taiyuan, China

**Keywords:** alternative splicing, hepatocellular carcinoma, hepatocellular carcinoma (HCC), iRIP-seq, SNRPA

## Abstract

**Background:**

Small Nuclear Ribonucleoprotein Polypeptide A (SNRPA), an RNA-binding protein associated with HCC survival, emerges as a key regulator requiring mechanistic study.

**Methods:**

To delineate SNRPA’s functions, we generated SNRPA-overexpressing HepG2 cells and performed RNA-seq to identify SNRPA-regulated transcripts and alternative splicing events(RASEs). Improved RNA Immunoprecipitation Sequencing (iRIP-seq) mapped RNA interactomes, combined with integrative bioinformatics to define SNRPA-bound RNAs and splicing targets.

**Results:**

RNA-seq revealed extensive transcriptomic alterations driven by SNRPA overexpression, including 498 differentially expressed genes(DEGs) and 2316 RASEs. iRIP-seq identified a subset of SNRPA-bound transcripts that also exhibited changes in expression and/or alternative splicing upon SNRPA overexpression, and many of these genes have been previously implicated in hepatocellular carcinoma (HCC)-related biological processes. Among the 498 DEGs identified, 12 RNA-binding proteins (RBPs) with known roles in HCC were found to be regulated by SNRPA overexpression, including PCBP2, HNRNPH1, and EIF4A2; these RBPs are involved in alternative splicing, mRNA stability and translational regulation, and their dysregulation further expands the regulatory network of SNRPA in HCC. Functional annotation of these overlapping genes indicated enrichment in pathways related to RNA processing, transcription-related processes, and cell division. SNRPA may play dual roles in RNA transcript binding and the regulation of splicing, particularly in pathways associated with tumorigenesis and cellular proliferation.

**Conclusions:**

These findings suggest that SNRPA overexpression is associated with HCC progression-related transcriptomic and splicing alterations, and may be involved in HCC progression through regulating the expression of DEGs and the splicing patterns of carcinogenic targets, with direct causal effects requiring further functional validation.

## 1 Introduction

Liver cancer represents a major global health burden, with rising incidence rates worldwide (1). Hepatocellular carcinoma (HCC), accounting for approximately 85% of primary liver malignancies (2), is characterized by aggressive progression and limited therapeutic efficacy in advanced stages. Despite advances in multimodal treatment approaches, late-stage diagnosis remains pervasive, contributing to persistently high mortality rates that mirror disease incidence (3, 4). Established etiological drivers include chronic hepatitis B/C viral infections, metabolic dysfunction-associated steatohepatitis (MASH), aflatoxin exposure, and lifestyle factors such as alcohol consumption and obesity (5). Molecular pathogenesis involves the progressive accumulation of genomic and epigenomic alterations, beginning in preneoplastic lesions and evolving through dysplastic nodules to overt HCC. The extensive genomic heterogeneity observed across HCC tumors underscores a complex evolutionary process driven by clonal selection of mutations disrupting key oncogenic pathways, including cell cycle regulation, chromatin remodeling, and Wnt/β-catenin signaling (6).

Recently, RNA-binding proteins (RBPs) have emerged as critical regulators in disease pathogenesis, particularly through their involvement in post-transcriptional gene regulation. These versatile proteins, often functioning within ribonucleoprotein complexes, mediate essential RNA processing events in eukaryotic cells - including mRNA stability maintenance, polyadenylation, splicing, translational control, and subcellular localization - through dynamic interactions with both coding and non-coding RNAs as well as protein partners (7, 8). Emerging evidence highlights their significant implications in hepatocellular carcinoma (HCC) progression. Notably, Feng et al. (9) demonstrated that RNA-Binding Protein 43(RBM43)deficiency accelerates hepatic carcinogenesis by modulating Cyclin B1 expression, while ribosomal protein S3(RPS3) overexpression promotes hepatocarcinogenesis through posttranscriptional upregulation of silent information regulator 1(SIRT1) (10). The oncogenic RBM3 has been shown to drive HCC cell proliferation via SCDcircRNA2 regulation (11), and other RBPs, including Poly(RC) Binding Protein 1(PCBP1) (12) and the LncRNA Metastasis Associated Lung Adenocarcinoma Transcript 1(MALAT1) (13) appear to influence HCC development through alternative splicing mechanisms. Although these studies underscore the multifaceted roles of RBPs in HCC pathogenesis, the comprehensive understanding of their regulatory networks - particularly regarding context-dependent functions, downstream effectors, and therapeutic potential - remains incomplete. Further systematic investigations are required to elucidate the complex RBP-mediated regulatory circuitry in HCC and identify novel diagnostic biomarkers or therapeutic targets.

Small nuclear ribonucleoprotein polypeptide A (SNRPA), a 282-amino-acid protein (~34 kDa), contains two canonical RNA recognition motifs (RRMs) and an N-terminal RNA-binding domain critical for its interaction with U1 snRNA (14, 15). Structural analyses reveal that specific amino acid residues proximal to these domains mediate sequence-specific RNA binding, particularly recognizing the AUUGCAC motif, which facilitates its essential role in pre-mRNA alternative splicing (14). Intriguingly, SNRPA also exhibits autoregulatory capacity through conserved 3’UTR interactions that coordinate *in vivo* cleavage and polyadenylation processes (16).

Emerging evidence positions SNRPA as a key player in tumorigenesis. Comparative tissue analyses demonstrate significant SNRPA overexpression in gastric carcinoma specimens relative to adjacent normal mucosa (p<0.01) (17). Suggesting its potential utility as both a diagnostic biomarker and therapeutic target. In hepatocellular carcinoma (HCC), SNRPA overexpression correlates strongly with microvascular invasion (MVI) status. Mechanistically, SNRPA drives epithelial-mesenchymal transition(EMT-like) phenotypic transitions in HCC cells by activating the NOTCH1/Snail signaling axis, thereby promoting metastatic dissemination *in vitro* and *in vivo* (18). Orthotopic xenograft models further confirm that SNRPA knockdown substantially reduces lung metastasis incidence (p<0.001) (18).

To systematically investigate SNRPA’s contribution to hepatocellular carcinoma (HCC), we established an SNRPA-overexpression (SNRPA-OE) model in HepG2 cells via lentiviral transduction (empty vector as control). Combining RNA sequencing (RNA-seq) with SNRPA-specific antibody-based individual-nucleotide resolution crosslinking and immunoprecipitation (iRIP-seq), we mapped genome-wide SNRPA-RNA interactions and their functional consequences. Integrative analysis revealed SNRPA’s dual regulatory role in (1) modulating oncogenesis-related gene expression programs and (2) orchestrating alternative splicing (AS) events critical for malignant transformation.

## 2 Materials and methods

### 2.1 Data collection and processing of tumor sample information

The Sequence Read Archive (SRA) was accessed to obtain publicly available sequence data files. The ‘fastq-dump’ utility was used to convert the SRA files into fastq format. The FASTX-Toolkit (version 0.0.13; http://hannonlab.cshl.edu/fastx_toolkit/) was used to initially conduct data processing. FastQC (https://www.bioinformatics.babraham.ac.uk/projects/fastqc/) was employed for quality evaluation.

### 2.2 Construction of an SNRPA-overexpression model in HepG2 cells

The human hepatocarcinoma cells(HepG2) cell line (CL-0103, Procell Life Science and Technology Co., Ltd., China) was categorized into two groups: the SNRPA -OE cells and normal cells (NC) groups, with three biological replicate samples in each group. All cells were cultured under standard conditions. The SNRPA-OE group received an overexpression SNRPA lentivirus [(Genepharma, Suzhou, China; Transcript information: NM_004596.5, lentiviral vector: LV5 (EF- 1a/GFP/Puro/Amp)]. The HepG2 cell line (CL-0103, Procell Life Science & Technology Co., Ltd., China) was maintained at 37°C in a 5% CO2 environment using MEM/F12 (PM150410, Procell Life Science and Technology Co., Ltd., China) supplemented with 10% fetal bovine serum (10091148, Gibco, China), along with 100 µg/mL streptomycin and 100 U/mL penicillin (SV30010, Hyclone, USA). HepG2 cells were infected with lentivirus at a multiplicity of infection of 100, and stable cell lines were selected using 1 µg/mL puromycin. Subsequently, these cells were collected for RT-qPCR and Western blotting analyses.

### 2.3 Proliferation experiment for HepG2 cells

Cell counting kit-8 (CCK-8, 40203ES76, Yeasen, Shanghai, China) was employed to conduct cell proliferation assays. The control and experimental groups were treated with appropriate blank controls. The blank control refers to the culture medium with CCK-8 solution only (without any HepG2 cells), which was used to eliminate the background absorbance of the reagents themselves in the optical density measurement. After incubating for 0, 24, 48, and 72 h, the culture medium was supplemented with 10 μl CCK-8 solution and incubated for an additional 3 h. At 450 nm, a Microplate Reader (ELX800, Biotek, USA) was utilized to measure cell optical density. The following formula was used to calculate cell proliferation rate:

Proliferation rate = (OD of experimental sample − OD of blank)/(OD of control – OD of blank) × 100%.

### 2.4 Western blot analysis of SNRPA protein levels

HepG2 cells were lysed by employing a RIPA buffer (PR20001, Proteintech, China) supplemented with protease inhibitors (4693116001, Sigma, USA). After boiling, the cell samples were analyzed using SDS-PAGE, transferred to a PVDF membrane, and subsequently blocked. The membranes were exposed to primary antibodies (anti-FLAG; 1:1000 dilution; mouse-derived; catalog number 66008-3-IG from Proteintech) and Actin (1:1000; rabbit-derived; 20536-1-AP, ThermoFisher). Subsequently, the membranes were incubated with horseradish peroxidase-conjugated secondary antibody (anti-mouse; 1:5000; AS003, ABclonal, China). Chemiluminescence visualization of the membranes was achieved using an ECL reagent (P0018FM, Beyotime, China).

### 2.5 Preparation and analysis of RNA-seq data from HepG2 cells

TRIZOL technique was utilized to extract total RNA, following the procedure outlined by Chomczynski et al. (19). After quantity and quality assessment of the purified RNA, the RNA-seq library was prepared using 2μg of total RNA, following the manufacturer’s guidelines for high-throughput sequencing. Each group (SNRPA-OE and NC) was prepared with three independent biological replicates. The cells used for RNA-seq were cultured to 70-80% confluency under standard conditions (37°C, 5% CO2) without additional stimulation, and total RNA was extracted at 48 h after lentiviral transfection when SNRPA overexpression was stably maintained. The PCR products were enriched, quantified, sequenced, and trimmed using the Novaseq 6000 sequencer (Illumina) and ASTX-Toolkit (Version 0.0.13). Quality-controlled reads were aligned to the GRCH38 genome using HISAT2 software (20). FPKM values were calculated based on uniquely mapped reads derived from gene sequencing. To identify DEGs, the DESeq2 package from R Bioconductor was used (21). Raw read counts were used as the input for DESeq2 for differential expression analysis, following the standard recommended workflow. FPKM values were only calculated for auxiliary gene expression level description, while variance-stabilized transformation (VST) was performed on the raw count matrix for PCA analysis to eliminate the influence of library size and heteroscedasticity, which is more suitable for cross-sample statistical inference.

### 2.6 Alternative splicing analysis of HepG2 cells

RASEs in the samples were identified and quantified by employing the ABLas pipeline, following established methods (22). For differential splicing analysis, the relative splicing levels of the same AS event were compared between the SNRPA-OE and NC groups using Student’s t-test across biological replicates, as implemented in the ABLas analysis workflow. Events with P ≤ 0.05 were considered regulated alternative splicing events (RASEs) in the initial differential screening. Because the initial splice-junction and AS-event catalogs were generated at the discovery stage, the detected novel junctions and novel AS events included candidates identified prior to stringent biological interpretation. Therefore, the proportions of novel junctions or AS events in the raw catalog were not interpreted as the proportions of high-confidence functional splicing events in downstream analyses. Briefly, PSI values were calculated within the ABLas pipeline using junction read counts, and Student’s t-test was applied to compare PSI values between groups. This approach provides an initial screening of differential splicing events but does not explicitly model junction-level uncertainty or replicate variability in a statistical framework. RASEs were defined in two steps: initial filtering by P ≤ 0.05, followed by stringent filtering using |ΔPSI| ≥ 0.1, FDR ≤ 0.05, and minimum junction read support.

### 2.7 Functional enrichment analysis

The KOBAS 2.0 server was used to investigate the enriched functions of DEGs and regulated alternative splicing genes (RASGs) via Gene Ontology (GO) terms analysis (23). Term enrichment was assessed by conducting a hypergeometric test, and the false discovery rate was controlled by applying the Benjamini-Hochberg procedure.

### 2.8 iRIP-seq preparation and data analysis

HepG2 cells were UV-crosslinked at 400 mJ/cm² and then lysed under RNase-free conditions. Cell lysates were subjected to controlled RNA digestion prior to immunoprecipitation. Briefly, lysates were first treated with RQ I RNase and subsequently with MNase, and the digestion was terminated by EDTA addition. The clarified lysates were then incubated overnight at 4 °C with anti-FLAG antibody (Sigma, F7425) or control IgG antibody, followed by capture of the immunocomplexes using protein A/G Dynabeads (Thermo Scientific). After sequential washing, the RNA-protein complexes were eluted, and proteinase K (Sangon Biotech) was added to both the 10% input samples and the immunoprecipitated samples at a final concentration of 1.2 mg/mL to digest proteins and release the associated RNA. The recovered RNA was purified and used for cDNA library construction with the KAPA RNA Hyper Prep Kit (KAPA, KK8544).

For data analysis, sequencing reads were aligned to the reference genome using HISAT2, and only uniquely mapped reads were retained after removal of PCR duplicates. Binding peaks were identified using Piranha and ABLIRC. Peak calling was performed with a false discovery rate (FDR) cutoff of 0.05, and the minimum peak length was set to 50 bp. For replicate handling, only peaks consistently detected in both biological replicates were retained for subsequent analysis to ensure the reliability of binding sites. In downstream analysis, the input samples were used as the baseline control for abundance comparison, and peaks enriched in the immunoprecipitation group relative to input were retained as SNRPA-associated binding peaks. Based on these peaks, putative target genes were assigned, and enriched binding motifs were identified using HOMER. The input sample RNA sequences were used as the background dataset for HOMER motif enrichment analysis, and GC bias correction was applied during the analysis process to reduce the impact of genomic GC content differences on motif identification results. Because the present iRIP-seq analysis was performed at the peak/region level, the resulting data were interpreted as providing transcript- or region-level evidence of SNRPA-associated RNA binding rather than exact nucleotide-resolution binding sites (24).

### 2.9 Reverse transcription qPCR validation of RASEs

cDNA synthesis was conducted following standard protocols. In addition, RT-qPCR was conducted on the QuantStudio5 using Hieff™ qPCR SYBR^®^ Green Master Mix (Low Rox Plus; YEASEN, China). The primers employed are listed in **Supplementary Table 1**. The qRT-PCR reaction conditions were as follows: pre-denaturation at 95°C for 5 min, followed by 40 cycles of denaturation at 95°C for 10 s and annealing/extension at 60°C for 30 s; a melting curve analysis (60-95°C) was performed after amplification to verify primer specificity. The Hieff qPCR SYBR^®^ Green Master Mix (Low Rox Plus, YEASEN) was used for the reaction, with a final reaction volume of 20 μL (10 μL master mix, 0.4 μL each primer (10 μM), 2 μL cDNA template, 7.2 μL nuclease-free water). Subsequently, the concentration of each transcript was normalized to the GAPDH mRNA level using the 2- ΔΔCT method (25).

## 3 Results

### 3.1 Transcriptome-wide identification of dysregulated RNA-binding protein genes in hepatocellular carcinoma clinical specimens

**Figure 1:**
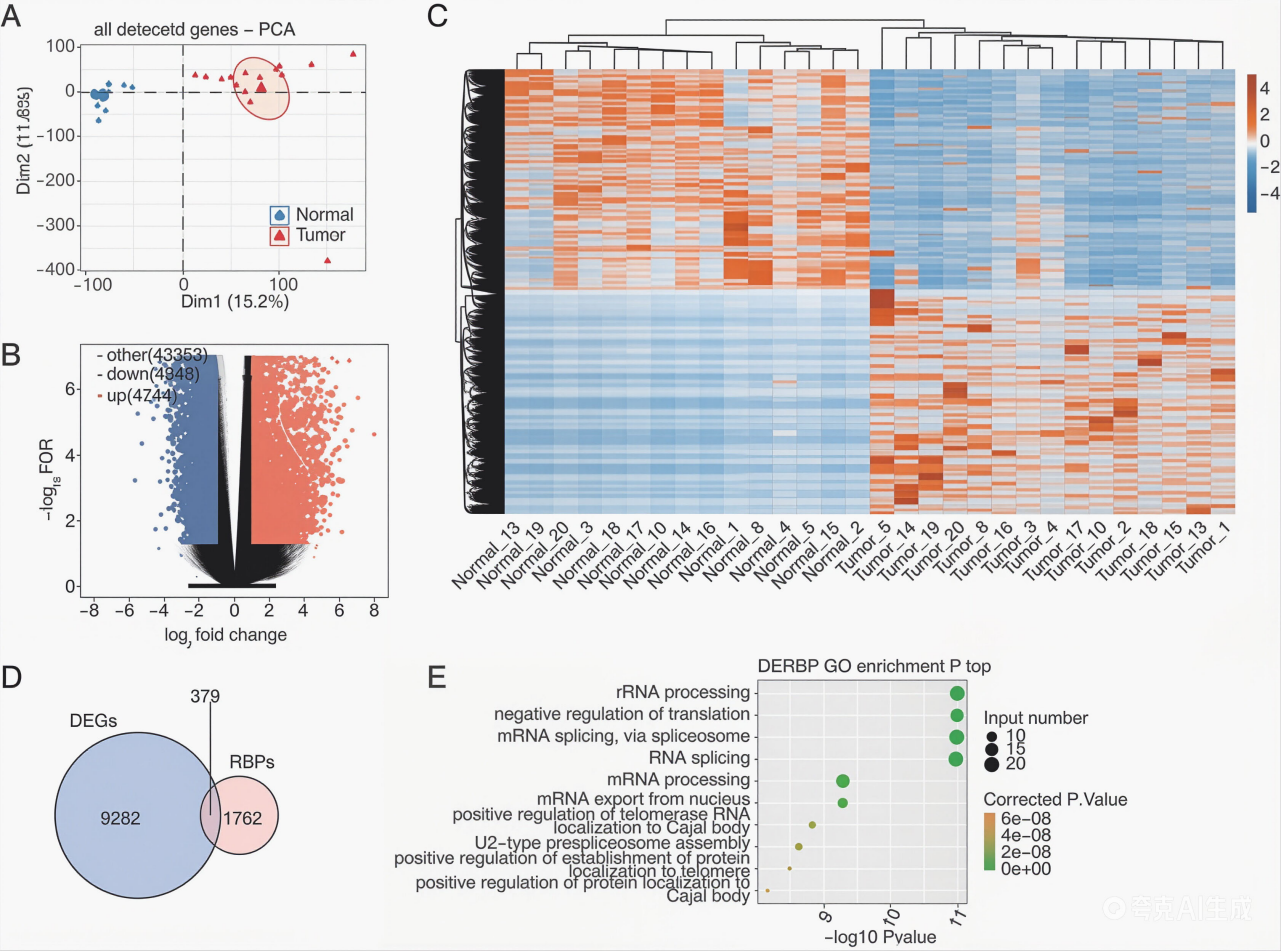
Differential gene expression analysis of the GSE77509 HCC cohort. **(A)** Principal component analysis (PCA) based on variance-stabilized transformed (VST) expression values of all detected genes. Confidence ellipses indicate the tumor and adjacent normal groups. **(B)** Volcano plot showing differentially expressed genes (DEGs) between tumor and adjacent normal tissues. Upregulated and downregulated genes are shown in red and blue, respectively. Differentially expressed RNA-binding protein (RBP) genes and core genes of key pathways (RNA processing, spliceosome, cell cycle) in the volcano plot correspond to the overlapping DERBPs of DEGs and RBP sets **(D)** and the significantly enriched GO biological processes of these DERBPs **(E)**, respectively, for easy reference and identification. **(C)** Hierarchical clustering heat map showing the expression patterns of all DEGs across tumor and adjacent normal samples. **(D)** Overlap between RNA-binding protein genes (RBPs) and DEGs. **(E)** Representative Gene Ontology (GO) biological process enrichment results for genes overlapping between the RBP set and the DEG set.

Transcriptome data from 15 HCC patients (GSE77509 dataset) were analyzed to identify DEGs and AS events between the tumor and adjacent normal tissues. Survival-correlated RBPs were further validated using TCGA-HCC cohorts. Principal component analysis (PCA) revealed distinct transcriptional profiles between tumor and normal tissues based on global gene expression patterns (**Figure 1A**). DEG analysis identified 4,744 upregulated and 4,948 downregulated genes (FDR <0.05, |log2FC| >1), visualized in a volcano plot (**Figure 1B**). Hierarchical clustering demonstrated consistent regulatory patterns between SNRPA-OE and control groups (**Figure 1C**).

Intersection analysis of DEGs with a curated RBP database (2,141 RBPs from four published studies (26–29). Identified 379 differentially expressed RBPs (DERBPs) (**Figure 1D**). Gene Ontology (GO) enrichment analysis revealed DERBPs were predominantly enriched in ribosomal RNA regulation, translational repression, spliceosome-mediated mRNA splicing, and mRNA processing (**Figure 1E**).

### 3.2 Alternative splicing alterations in GSE77509 identify SNRPA as an HCC-associated candidate RBP

**Figure 2:**
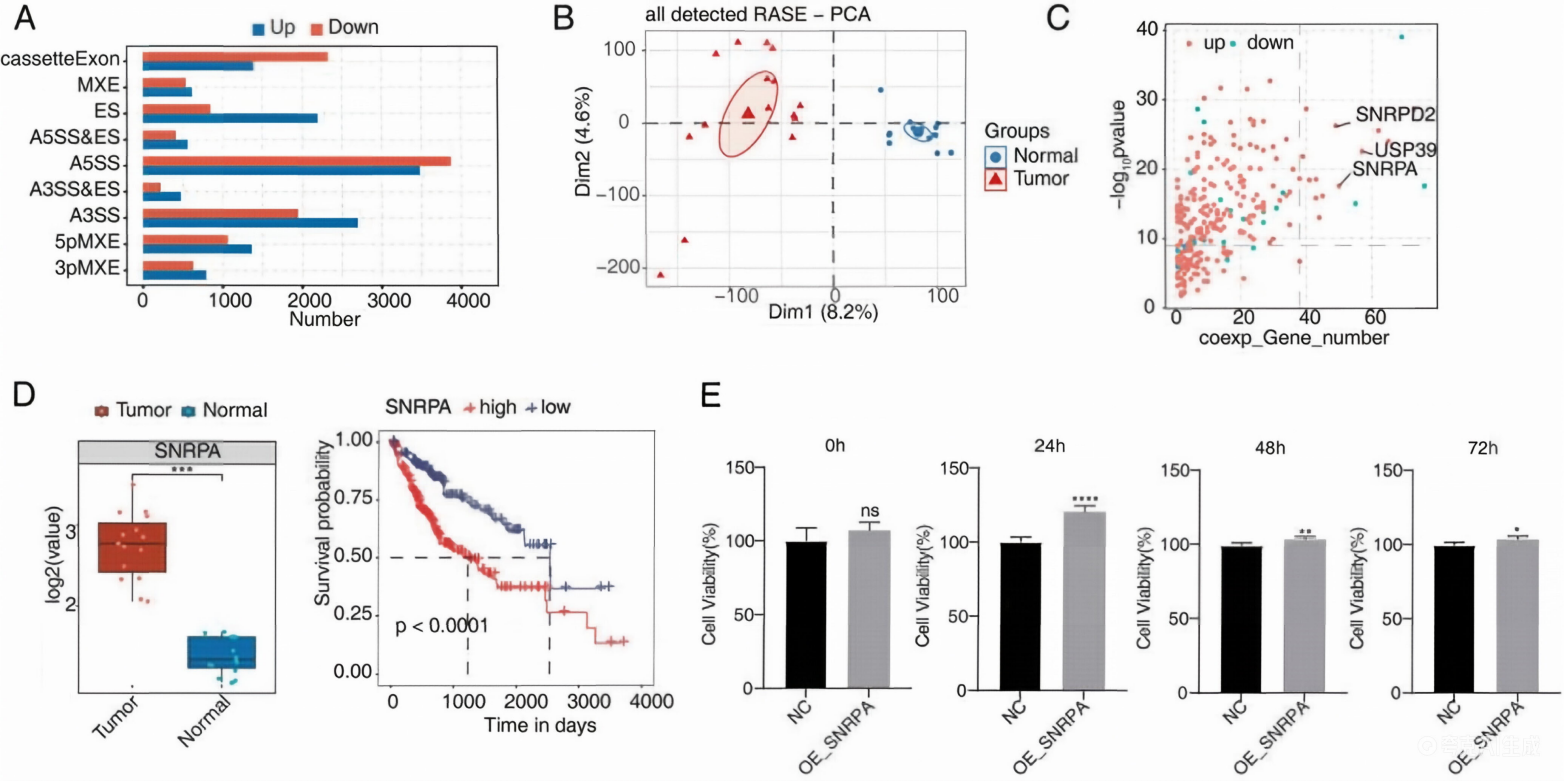
Alternative splicing analysis and prioritization of SNRPA in HCC. **(A)** Distribution of regulated alternative splicing events (RASEs) between tumor and adjacent normal tissue samples. **(B)** Principal component analysis (PCA) based on the Percent Spliced In (PSI) values of identified RASEs. **(C)** Co-expression analysis of differentially expressed RNA-binding proteins (DERBPs) and RASEs. Red points indicate upregulated DERBPs, and blue points indicate downregulated DERBPs. Significant co-expression pairs were identified based on Pearson correlation analysis with the following criteria: absolute correlation coefficient ≥ 0.85, and Benjamini-Hochberg-adjusted P-value (FDR) ≤ 0.01. To assess the robustness of correlation estimates in the limited sample size (n = 15), permutation analysis (1,000 permutations) was performed, and only pairs with permutation-based P ≤ 0.05 were retained as significant. **(D)** Expression level and survival analysis of SNRPA in LIHC cohorts. Left: expression of SNRPA in tumor and adjacent normal tissues. Right: overall survival analysis according to SNRPA expression level. **(E)** CCK-8 assay showing the viability of HepG2 cells following SNRPA overexpression at 24, 48, and 72 h. Statistical significance was evaluated using Student’s t-test. ***P < 0.001, **P < 0.01, *P < 0.05.

Alternative splicing profiles in tumor and adjacent normal samples were compared using the GSE77509 dataset and identified nine categories of regulated alternative splicing events (RASEs) in tumor samples. Among these RASEs, four types, including cassette exon, exon skip (ES), alternative 5’ splice site (A5SS) and alternative 3’ splice site (A3SS) exhibited a relatively high prevalence, with A3SS and A5SS being the most frequent (**Figure 2A**). PCA further verified a substantial difference in the occurrence of AS events between the tumor and normal groups (**Figure 2B**). Based on these findings, a DERBP protein interaction network was constructed (**Figure 2C**), which revealed modules comprising 272 key DERBPs that were significantly associated with AS events (correlation ≥0.85 or ≤-0.85, P < 0.05). Among the identified RBP genes, ubiquitin-specific peptidase 39 (USP39), SNRPA and Small Nuclear Ribonucleoprotein D2 Polypeptide (SNRPD2) had the largest number of co-expressed pairs involving differential AS events. Ultimately, SNRPA was selected as the target RBP gene linked to HCC due to its comprehensive significance.

To explore the expression profile of SNRPA and its prognostic significance in HCC, the GEPIA2 server (30) was used for gene expression analysis, whereas the KM-plotter tool (31) was employed for survival analysis. The gene expression data for SNRPA in tumor and adjacent normal tissues were retrieved from the TCGA-LIHC cohort via the GEPIA2 server, and the survival analysis data were obtained from the KM-plotter tool which integrates RNA-seq data from the TCGA-LIHC and GEO (GSE77509) cohorts for liver hepatocellular carcinoma. SNRPA expression was significantly higher in tumor tissues than in adjacent normal tissues (GEO dataset, **Figures 2D left**). Additionally, a moderate but significant increase was observed in adjacent normal tissues compared with normal liver tissues from healthy donors. Furthermore, survival analysis using the KM-plotter revealed that lower SNRPA levels were associated with longer overall survival (OS) in individuals with liver hepatocellular carcinoma (LIHC) (**Figures 2D right**). A proliferation assay performed after SNRPA overexpression demonstrated a significant increase in HepG2 cell proliferation, with cell viability peaking 24 h post-lentivirus transfection (**Figure 2E**). These results suggest that SNRPA may be associated with pro-proliferative effects in HCC cells and support its potential relevance to HCC progression. This conclusion is supported by two lines of evidence (1): CCK-8 assay showed a significant increase in proliferation of SNRPA-overexpressing HepG2 cells at 24/48/72 h (***P < 0.001, **Figure 2E**) (2); survival analysis revealed that high SNRPA expression was associated with reduced overall survival in LIHC patients (**Figure 2D**), consistent with previous reports that SNRPA promotes HCC metastasis via the NOTCH1/Snail pathway. In addition, elevated SNRPA expression was associated with reduced overall survival in the analyzed LIHC patient cohort.

### 3.3 Gene expression changes associated with SNRPA overexpression in HepG2 cells

**Figure 3:**
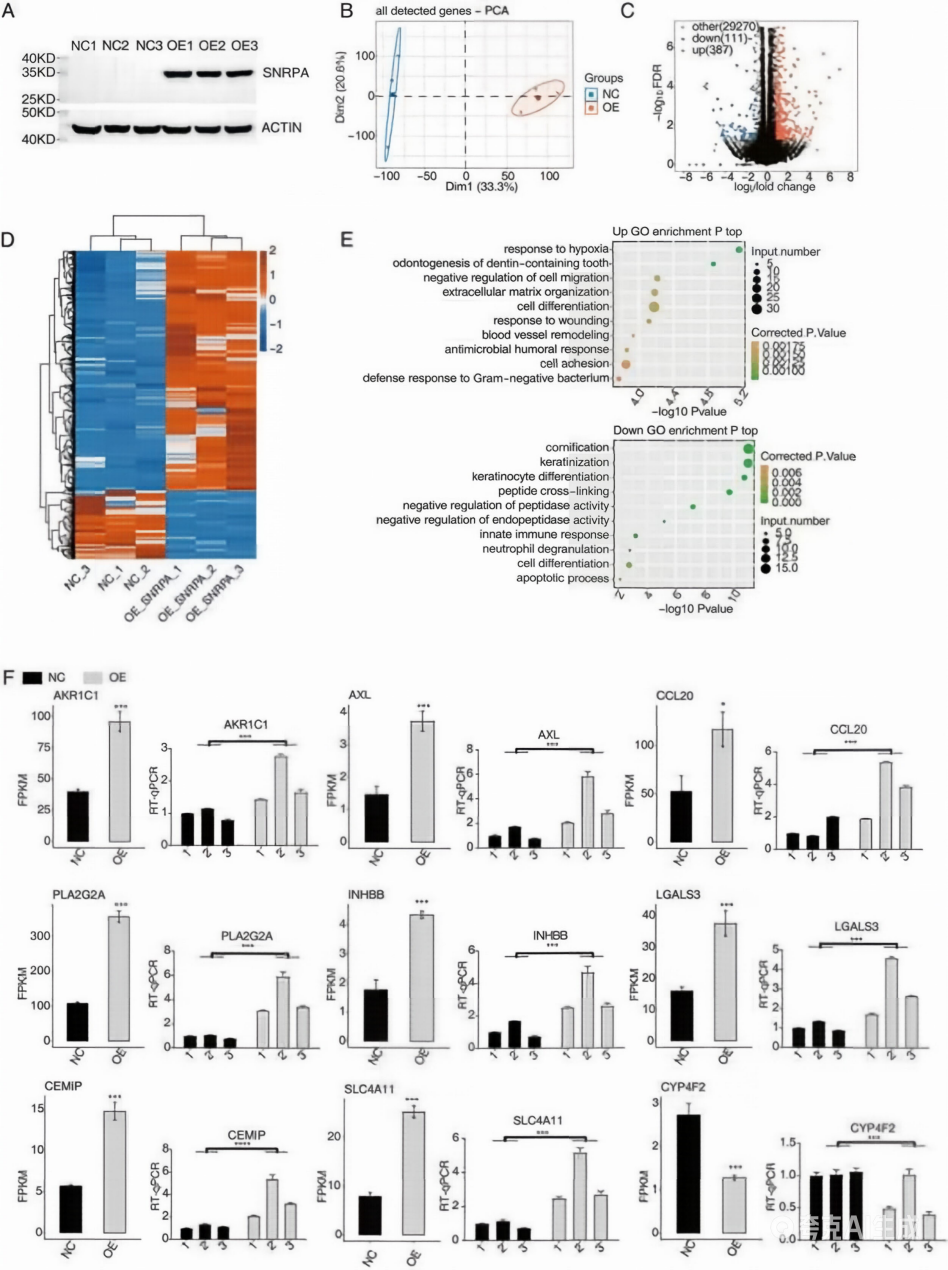
Gene expression changes associated with SNRPA overexpression in HepG2 cells. **(A)** Western blot analysis confirming successful SNRPA overexpression in HepG2 cells. **(B)** Principal component analysis (PCA) based on variance-stabilized transformed (VST) expression values of all detected genes. Confidence ellipses indicate the SNRPA-OE and NC groups. **(C)** Volcano plot showing differentially expressed genes (DEGs) between the SNRPA-OE and NC groups. **(D)** Hierarchical clustering heat map showing the expression patterns of DEGs in the SNRPA-OE and NC groups. **(E)** Representative Gene Ontology (GO) biological process enrichment results for upregulated genes (left) and downregulated genes (right). **(F)** RT-qPCR validation of representative DEGs identified by RNA-seq. Bar plots show the mean relative expression ± SD from three biological replicates (each with three technical replicates). Statistical significance was determined by two-tailed Student’s t-test: ***P < 0.001, ****P < 0.0001.

To further investigate the relationship between SNRPA overexpression and transcriptomic changes, we established an SNRPA-overexpressing HepG2 cell model. Western blot analysis demonstrated a significant increase in SNRPA protein expression in transfected cells compared to controls (**Figure 3A**), validating the successful generation of the SNRPA-overexpressing cellular model. To investigate genome-wide transcriptional alterations, we performed RNA sequencing on cDNA libraries prepared from SNRPA-OE and control cells. High-throughput sequencing yielded approximately 73 million paired-end raw reads per sample, with 72 million high-quality reads retained after adapter trimming and quality filtering (Q30 > 90%). HISAT2 alignment against the GRCh38 reference genome showed 96.4% overall mapping efficiency, with 94.2% of reads uniquely mapped (**Supplementary Table 2**).

PCA of variance-stabilized transformed (VST) expression values revealed distinct clustering of SNRPA-OE samples along the first principal component (explaining 78% variance), indicating marked transcriptomic differences associated with SNRPA overexpression (**Figure 3B**). Differential expression analysis using DESeq2 (|log2FC| ≥1, adjusted P-value < 0.05) identified 498 significantly regulated genes - 387 upregulated and 111 downregulated in SNRPA-OE cells (**Figure 3C**; **Supplementary Table 3**). Hierarchical clustering of these DEGs demonstrated high intra-group reproducibility (Pearson’s r >0.95 between replicates) and clear inter-group separation (**Figure 3D**).

GO enrichment analysis revealed distinct biological processes modulated by SNRPA overexpression (**Figure 3E**). Upregulated genes exhibited significant enrichment (FDR-adjusted P< 0.05) in biological processes previously reported to be relevant to HCC biology: hypoxia response (32, 33), extracellular matrix organization (34), cell differentiation (35), wound-healing response (36), vascular remodeling (37), and cell adhesion (38). In contrast, the downregulated DEGs exhibited notable enrichment in pathways such as keratinocyte division, glue cross-linking, negatively regulated peptidase activity, innate immune response, medium sexual granulocyte degranulation, apoptosis, and other biological function pathways. The observed suppression of protease regulatory pathways corroborates previous findings by Huang et al. (39), who demonstrated that impaired peptidase activity in HCC models disrupts caspase-dependent apoptosis through aberrant phosphorylation of BCL-2 family proteins. Notably, the concurrent downregulation of neutrophil-mediated immunity may be relevant to immune-related alterations in HCC, although the current data do not establish a direct role for SNRPA in immune evasion.

Based on the expression levels of DEGs in the SNRPA-OE group, several genes were identified as relevant to liver cancer, including Galectin 3(LGALS3) (40), Phospholipase A2 Group IIA(PLA2G2A) (41), Aldo-Keto Reductase Family 1 Member C1(AKR1C1) (42), and C-C Motif Chemokine Ligand 20 (CCL20) (43), which ranked among the top 40. Furthermore, Inhibin Subunit Beta B(INHBB) (44), LGALS3, and AXL Receptor Tyrosine Kinase(AXL) (45) are associated with the cell differentiation pathway. The expression levels of the six aforementioned genes were notably upregulated. In contrast, Cytochrome P450 Family 4 Subfamily F Member 2 (CYP4F2) exhibited marked downregulation, suggesting potential metabolic reprogramming effects requiring mechanistic investigation. Reverse transcription-quantitative polymerase chain reaction (RT-qPCR) validation demonstrated strong concordance with RNA-seq data (**Figure 3F**), with LGALS3 showing the most pronounced overexpression and CYP4F2 displaying significant suppression. These findings indicate that SNRPA overexpression is associated with transcriptomic alterations in HepG2 cells, with altered expression of multiple genes that have been previously linked to HCC-related biological processes.

### 3.4 Alternative splicing changes associated with SNRPA overexpression in HepG2 cells

**Figure 4:**
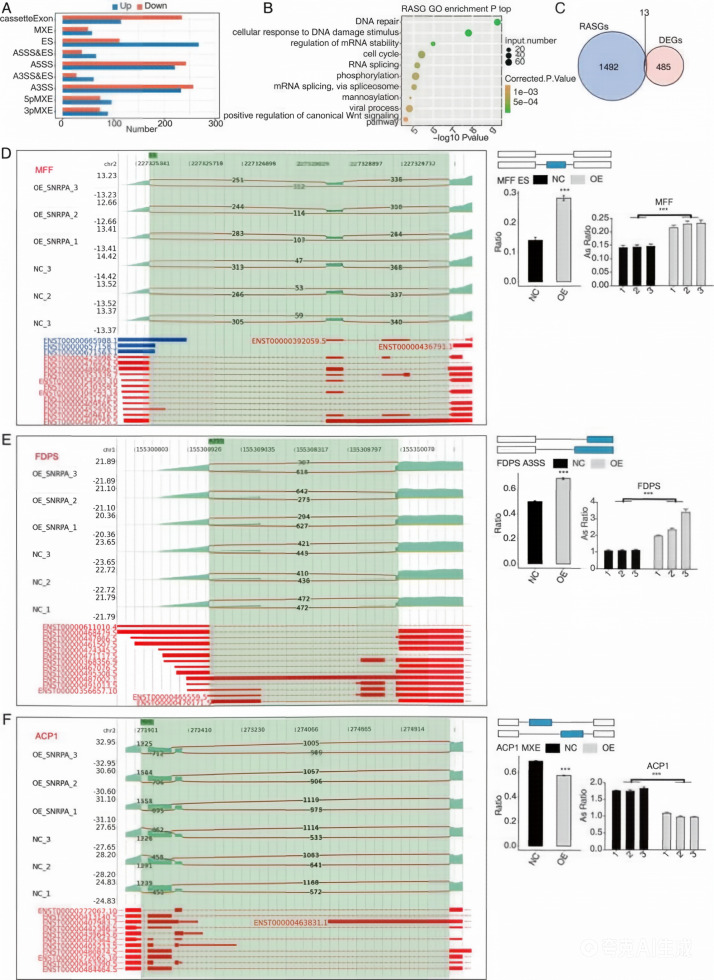
Alternative splicing changes associated with SNRPA overexpression in HepG2 cells. **(A)** Distribution and classification of regulated alternative splicing events (RASEs) identified between the SNRPA-OE and NC groups. **(B)** Representative Gene Ontology (GO) biological process enrichment results for regulated alternative splicing genes (RASGs). **(C)** Venn diagram showing the overlap between regulated alternative splicing genes (RASGs) and differentially expressed genes (DEGs). **(D)** Representative alternative splicing event in MFF associated with SNRPA overexpression. Left: sashimi plot showing junction read distribution and transcript structure, which illustrates the splicing pattern changes but does not confirm direct regulatory effects of SNRPA on these splicing events. Right: schematic diagram of the RASE, with RNA-seq quantification and RT-qPCR validation shown below. RT-qPCR validation of the representative RASE in MFF associated with SNRPA overexpression. Bar plots show relative splicing ratio with error bars representing the standard deviation (SD) from three biological replicates (each with three technical replicates). Statistical significance was determined by two-tailed Student’s t-test: ***P < 0.001. **(E)** Representative alternative splicing event in FDPS associated with SNRPA overexpression, which illustrates the splicing pattern changes but does not confirm direct regulatory effects of SNRPA on these splicing events. **(F)** Representative alternative splicing event in ACP1 associated with SNRPA overexpression, which illustrates the splicing pattern changes but does not confirm direct regulatory effects of SNRPA on these splicing events.

To investigate alternative splicing changes associated with SNRPA overexpression in HCC cells, we performed a transcriptome-wide AS analysis using the RNA-seq dataset. The HISAT2 aligner identified 390,716 splice junctions across all samples, including 196,292 junctions not present in the reference annotation and 194,424 annotated junctions (**Supplementary Table 4**). Because this initial junction catalog was generated at the detection stage, the proportion of novel junctions should be interpreted cautiously and not regarded as the proportion of high-confidence functional splice sites. Further analysis using the ABLas pipeline identified 103,706 alternative splicing (AS) events across all samples, including 49,961 annotated events and 53,745 putative novel events. Using Student’s t-test to compare relative splicing levels between the SNRPA-OE and NC groups, we identified 2,316 significantly altered regulated alternative splicing events (RASEs) at P ≤ 0.05. Stringent filtering criteria for RASEs were additionally applied: the absolute change in Percent Spliced In (ΔPSI) value was ≥ 0.1, the FDR corrected by Benjamini-Hochberg procedure was ≤ 0.05, and each splice junction had at least 10 uniquely mapped reads for support in all biological replicates. These events were classified into nine canonical splicing categories (**Supplementary Table 5**), with cassette exon inclusion/exclusion and mutually exclusive exon events representing the major event types (**Figure 4A**). A subset of differential splicing events occurred in genes previously reported to be associated with HCC-related biological processes, including epithelial-mesenchymal transition and metabolic regulation. Overall, these results indicate that SNRPA overexpression is associated with widespread alterations in transcript isoform usage in HepG2 cells, including both annotated and putative novel splicing events.

GO enrichment analysis of regulated alternative splicing genes (RASGs) revealed significant enrichment in pathways related to DNA repair, cellular response to DNA damage, regulation of mRNA stability, cell cycle control, RNA splicing-related processes, and Wnt signaling (**Figure 4B**).To identify genes showing both expression changes and alternative splicing alterations in association with SNRPA overexpression, we compared the DEG and RASG datasets. This analysis identified 13 genes common to DEGs and RASGs (**Figure 4C**). Subsequently, genes associated with HCC were prioritized based on standard criteria: the smallest FDR-corrected P-values, the highest read counts for splice junctions, and the most significant ΔPSI values (46). Ultimately, six events with significant differences were identified, including Mitochondrial Fission Factor (MFF), which demonstrated a strong correlation between its overexpression and poor prognosis in patients with HCC (47); Farnesyl Diphosphate Synthase(FDPS), which is associated with the prognosis of cholesterol metabolism in HCC, providing valuable insights into disease progression (48); Acid Phosphatase(ACP), which exhibits frequent hypermethylation in HCC events (49); Enoyl-CoA Hydratase Domain Containing 2(ECHDC2), whose overexpression is associated with a favorable prognosis in patients with HBV-associated conditions (46); Poly(RC) Binding Protein 2(PCBP2), which affects the proliferative response of cancer nodules, as noted by Li, Zhao, Lin, Liu and Cheng (50); and the repression of GNAS Complex Locus (GNAS), which leads to the attenuation of IL-6 expression induced by Lipopolysaccharides (LPS)through the inhibition of Signal Transducer And Activator Of Transcription 3(STAT3)activation in HCC cells (51). To validate representative RASGs associated with SNRPA overexpression, RT-qPCR experiments were performed, revealing a strong correlation between the six RASEs and transcriptome analysis results. The SNRPA-OE group exhibited higher AS ratios for MFF, FDPS, ECHDC2, PCBP2, and GNAS, as confirmed by RNA-seq and qPCR analysis, compared with the NC group. However, ACP1 exhibited opposing effects (**Figures 4D–F**; **Supplementary Figures 1**, **2B**).

### 3.5 iRIP-seq identifies SNRPA-associated transcripts in HepG2 cells

**Figure 5:**
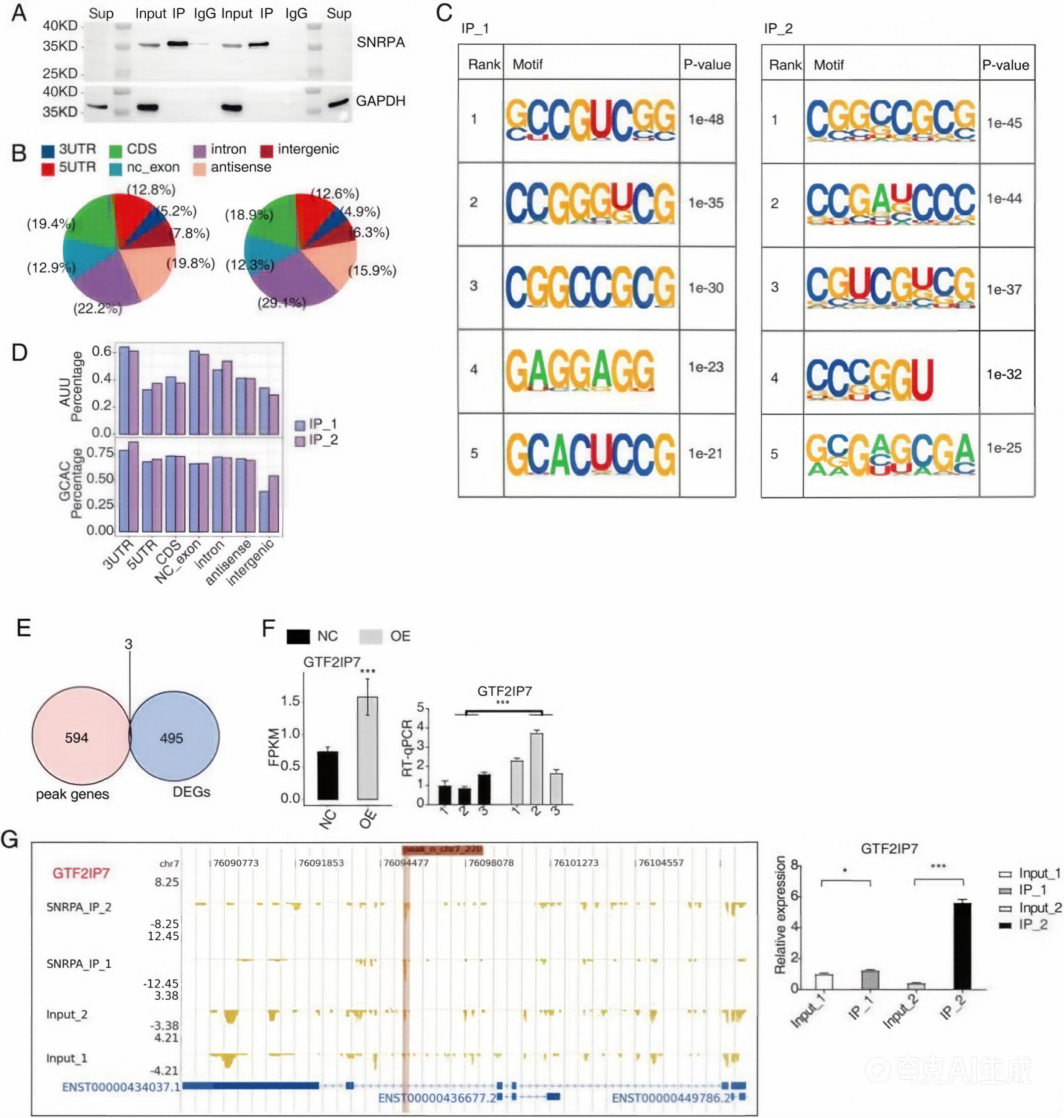
iRIP-seq profiling of SNRPA-associated transcripts in HepG2 cells. **(A)** Western blot analysis of SNRPA immunoprecipitation in two biological replicates. “sup” indicates the supernatant after immunoprecipitation, “lan2” indicates the protein marker, and “input” represents the control sample without immunoprecipitation. “IP” denotes immunoprecipitation with anti-SNRPA antibody, whereas “IgG” denotes the IgG immunoprecipitation control. **(B)** Genomic distribution of SNRPA-binding peaks in the two biological replicates. **(C)** Motif enrichment analysis of SNRPA-binding peaks identified in the two biological replicates. **(D)** Representative enriched sequence motifs identified in both iRIP-seq datasets. **(E)** Venn diagram showing the overlap between peak-associated genes and differentially expressed genes (DEGs). **(F)** Expression analysis and RT-qPCR validation of overlapping genes between peak-associated genes and DEGs. **(G)** Representative SNRPA-binding signal in GTF2IP7. Left: read density profile of the SNRPA-binding peak across the GTF2IP7 locus. Right: RIP-PCR/qRT-PCR validation of GTF2IP7 association with SNRPA. *P < 0.05, ***P < 0.001.

To profile SNRPA-associated RNAs in HepG2 cells, we performed iRIP-seq using anti-FLAG immunoprecipitation followed by high-throughput sequencing (iRIP-seq, see Materials and Methods) to identify transcripts interacting with this RBP. To ensure experimental reproducibility, two independent biological replicates were conducted. Western blot analysis confirmed efficient SNRPA immunoprecipitation, demonstrating antibody specificity in immunoprecipitated fractions compared to input controls (**Figure 5A**). High-quality sequencing libraries prepared from SNRPA-IP and matched Input samples generated comprehensive iRIP-seq datasets (see **Supplementary Table 6** for sequencing metrics).

Following adapter trimming and quality filtering, approximately 88.65% of processed reads showed accurate alignment to the GRCh38 reference genome using HISAT2 (alignment statistics in **Supplementary Table 7**). Although UV crosslinking and controlled nuclease digestion were incorporated into the iRIP-seq workflow, the downstream analysis was performed at the peak/region level; therefore, the resulting data were interpreted as providing transcript-/region-level binding evidence rather than exact nucleotide-resolution binding sites. Comparative genomic analysis revealed significant enrichment of uniquely mapped anti-SNRPA reads in 5’UTRs of immunoprecipitated samples relative to input controls (**Supplementary Table 8**), a particularly noteworthy observation given the methodological constraints of intact transcript analysis.

To identify the binding sites of SNRPA from the reads obtained through RIP-seq, ABLIRC, a recently released software previously described (22), was used. This software captures and compares overlapping tags with the highest degree of similarity to target genes. Using the Input sample as a baseline, peak calling was conducted on the reads removed from PCR duplication for the experimental sample, yielding 597 peaks. The peak-calling thresholds for iRIP-seq were set as follows: FDR ≤ 0.05, fold enrichment of IP over input ≥ 2, and only peaks detected in both biological replicates were retained. The relatively limited number of peaks is due to the strict replicate consistency requirement and high fold enrichment cutoff, ensuring the high confidence of the identified SNRPA-binding sites. As shown in (**Figure 5B**), the peaks’ genomic distribution associated with SNRPA in both SNRPA-IP samples demonstrated remarkable similarity, highlighting the high reproducibility of the samples. Furthermore, a significant concentration of peaks was observed in the 5’UTR and CDS regions. iRIP-seq data analysis also revealed that SNRPA binds to a variety of non-coding RNAs (ncRNAs), including 23 long non-coding RNAs (lncRNAs, e.g., MALAT1, NEAT1), 8 microRNA (miRNA) primary transcripts (pri-miRNAs), and 12 transfer RNAs (tRNAs). Among these, MALAT1 is a well-characterized HCC-associated lncRNA that modulates alternative splicing, suggesting that SNRPA may also regulate HCC progression through binding to ncRNAs, with detailed regulatory mechanisms to be further investigated. The motif analysis (**Figures 5C, D**) revealed that the binding motifs of SNRPA were strongly consistent across both experiments. Notably, SNRPA exhibited affinity toward the GCAC and AUU motifs of RNA, which aligns with the results observed in previous research (52). These motif results suggest that SNRPA may preferentially associate with AUUGCAC-containing RNA regions, including sites located in 5′ UTRs (53), however, the current data do not establish a direct effect on pre-mRNA processing or polyadenylation. A comprehensive gene overlap analysis was performed to further elucidate the potential genes involved in the regulation of SNRPA by DEGs in HepG2 cells. This analysis compared the 498 significant DEGs identified from the SNRPA-OE RNA-seq data with the peak genes detected in the iRIP-seq data. This analysis identified three upregulated genes, namely Cell Migration Inducing Hyaluronidase 1(CEMIP) (54), Solute Carrier Family 4 Member 11 (SLC4A11) (55), and General Transcription Factor IIi Pseudogene 7(GTF2IP7), which contain binding sites for SNRPA. As shown in the Venn diagram (**Figure 5E**), these genes have been reported to be relevant to HCC biology and may represent candidate SNRPA-associated transcripts showing concordant binding evidence and expression changes.

RT-qRCR experiments were conducted to confirm the accuracy of RNA-seq and iRIP-seq. The results of the three genes, CEMIP, SLC4A11 (**Figure 3F**), and GTF2IP7 (**Figure 5F**), indicated strong alignment with the results from the examination of RNA-seq data for transcriptome analysis. However, among the tested genes, RIP-PCR provided direct experimental support for SNRPA association only in the case of GTF2IP7. As shown in the left image of the IGV-sashimi plot, the binding site of SNRPA to the GTF2IP7 peak gene is visible. This finding was further supported by the RIP-PCR results, indicating the effective binding between SNRPA and GTF2IP7 (**Figure 5G**).

### 3.6 Candidate HCC-related splicing transcripts associated with SNRPA binding in HepG2 cells

**Figure 6:**
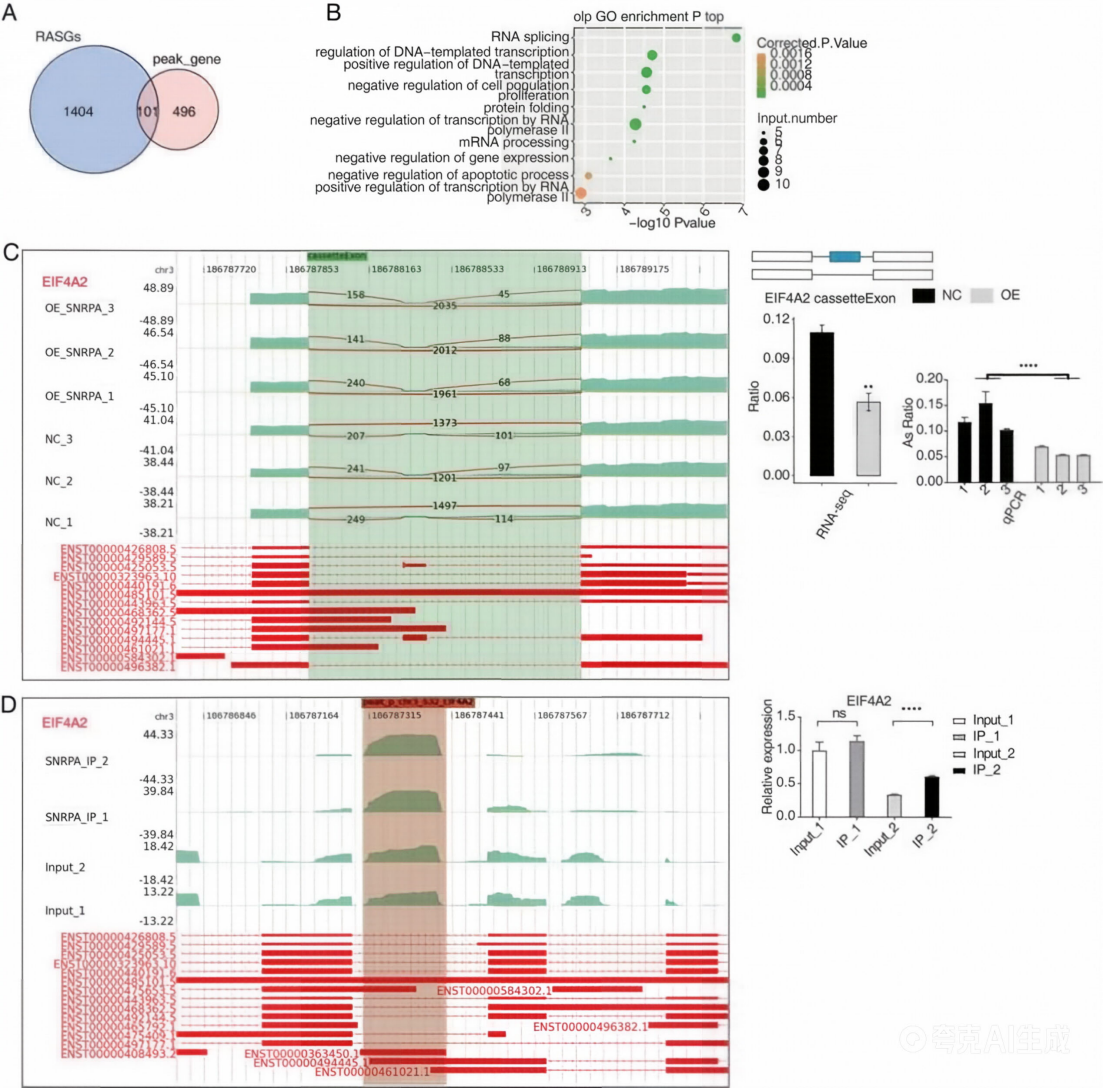
Candidate HCC-related splicing transcripts with SNRPA-binding evidence in HepG2 cells **(A)** Number of overlapping genes between peak-associated genes and regulated alternative splicing genes (RASGs). **(B)** Representative Gene Ontology (GO) biological process enrichment results for genes shared between the peak-associated gene set and the RASG set. **(C)** Representative alternative splicing event in EIF4A2 associated with SNRPA overexpression. Left: sashimi plot showing junction read distribution and transcript structure. Right: schematic diagram of the alternative splicing event, with RNA-seq quantification and RT-qPCR validation shown below. **(D)** Representative SNRPA-binding signal in EIF4A2. Left: read density profile of the SNRPA-binding peak across the EIF4A2 locus. Right: RIP-PCR/qRT-PCR validation of EIF4A2 association with SNRPA. **P < 0.01, ****P < 0.0001.

An overlap analysis was performed between the 597 peak genes corresponding to 1505 significantly differentially expressed RNA-associated splicing genes in RNA-seq data following SNRPA overexpression and the genes exhibiting peaks in both experiments from the iRIP-seq data. This analysis identified 101 overlapping genes between the peak genes and RASGs (**Figure 6A**), suggesting that these 101 RASGs contain SNRPA binding sites and exhibit substantial differences in alternative splicing levels. Analysis of the positional relationship between SNRPA-binding peaks and splice sites showed that 42% of the binding peaks were located within 500 bp of exon-intron boundaries, indicating a potential spatial proximity between SNRPA binding and alternative splicing regulation of these RASGs, although direct regulatory evidence requires further functional validation. The scatter plot (**Figure 6B**) demonstrates their roles in RNA splicing, DNA template transcription regulation, and negative cell division regulation. These results suggest that these genes may represent candidate transcripts whose splicing changes are associated with SNRPA in HepG2 cells. Validated genes were selected based on three core criteria (1): presence of SNRPA-binding peaks in iRIP-seq data (FDR ≤ 0.05, replicate consistency) (2); significant alternative splicing changes in RNA-seq data (ΔPSI ≥ 0.1, FDR ≤ 0.05) (3); previous reports implicating the genes in HCC tumorigenesis and progression. In this study, Heterogeneous Nuclear Ribonucleoprotein H1(HNRNPH1), Eukaryotic Translation Initiation Factor 4A2(EIF4A2), Protein Phosphatase 6 Regulatory Subunit 2(PPP6R2), and Fibronectin 1(FN1) are differential alternative splicing event-related genes of significant interest. The SNRPA-OE group exhibited a substantial decrease in the expression levels of EIF4A2 (**Figure 6C**), PPP6R2 (**Supplementary Figure 3A**), and FN1 (**Supplementary Figure 4A**) compared with the control group. Furthermore, a notable increase was observed in alternative splicing events for HNRNPH1 (**Supplementary Figure 3A**) and GNAS (**Supplementary Figure 2B**). These results closely align with expected outcomes. In addition, RIP-PCR experiments for EIF4A2 (**Figure 6D**), HNRNPH1 (**Supplementary Figure 3B**), and FN1 (**Supplementary Figure 4B**) revealed statistical differences, indicating the strong binding affinity of SNRPA to RNAs encoded by these three target genes.

## 4 Discussion

The present study suggests that SNRPA overexpression is associated with coordinated changes in gene expression and alternative splicing in HCC cells. All findings are based on an overexpression model; thus, future studies using SNRPA knockdown or knockout models are required to validate causality and exclude potential artificial effects associated with overexpression. Our findings support a potential role for SNRPA in linking RNA-binding activity with transcriptomic alterations relevant to oncogenic pathways, providing an initial framework for understanding its possible contribution to HCC progression.

The observed association between elevated SNRPA expression and reduced overall survival in LIHC patients is consistent with the increased proliferation observed in SNRPA-overexpressing HepG2 cells. Although the current data do not establish causality, this pattern may be related to transcriptomic changes associated with SNRPA overexpression, including 498 differentially expressed genes enriched in pathways involved in cell cycle regulation and DNA damage response.

The upregulation of differentiation regulators INHBB, LGALS3, and AXL in HCC drives tumor progression by maintaining progenitor-like cellular states through impaired differentiation (delayed maturation or aberrant trajectories). The concurrent downregulation of tumor suppressors CYP4F2 suggests SNRPA participates in a coordinated program to disable cellular checkpoints while activating proliferation signals. The identified 2316 RASEs reveals SNRPA’s genome-wide splicing regulatory capacity.

iRIP-seq analysis showed non-random distribution patterns of SNRPA-binding peaks, with 34.58% of peaks located in intronic regions and 24.75% in intergenic regions, indicating that SNRPA-associated RNAs were not restricted to protein-coding exons. We also observed enrichment of SNRPA-binding peaks in 5′ UTR regions, including peaks containing the AUUGCAC motif. These findings are consistent with selective RNA association by SNRPA, although they do not by themselves establish direct regulation of exon-intron boundary selection or specific splicing decisions.Integrated analysis of RNA-seq and iRIP-seq data identified three upregulated genes (GTF2IP7, CEMIP, and SLC4A11) that also showed SNRPA-binding signals. These genes may represent candidate SNRPA-associated targets linking RNA-binding evidence with expression changes in SNRPA-overexpressing cells.

In particular, CEMIP (KIAA1199) has been reported to be highly expressed in HCC samples and to be associated with tumor size, microvascular invasion, and poor survival (56, 57). Mechanistically, CEMIP enhances the EMT process and promotes HCC metastasis by modulating phosphorylation events involving the EGFR, STAT3, and ERK1/2 signaling pathways (58). As a core component of the U1 snRNP complex, SNRPA dysregulation may be associated with abnormal splicing patterns, including exon skipping events (59). This splicing change may drives pathogenic exon skipping in the CEMIP transcript, potentially promoting hepatocellular carcinoma progression through enhanced tumor cell migration mediated by abnormal hyaluronidase activity. In addition, SLC4A11 is upregulated in HCC tumor tissues and is activated by the β-catenin/TCF4 pathway, which results in increased ammonia emission and supports the progression of mutant HCC (60). These observations suggest a potential functional link among SNRPA, CEMIP, and SLC4A11 in HCC-associated signaling networks. However, the current data remain associative and do not establish a direct regulatory circuit, reciprocal activation, or causal relationship. This regulatory relationship may be relevant to EMT-related changes and metabolic reprogramming in HCC, although the current data do not establish a feedforward loop or a direct role in metastatic progression.

In addition, by integrating iRIP-seq data with variable splicing event data, a set of validated genes (EIF4A2, FN1, and hnRNPH1) finally caught our interest.EIF4A2, a member of the EIF4A family of eukaryotic initiation factors, is essential for the translation process (61). The translation initiation complex involving EIF4A2 regulates the translation rates of various mRNAs related to cancer promotion and serves as a key component within the regulatory framework of the PI3K/AKT/mTOR signaling transduction pathway (62). Previous studies have reported that EIF4AII is upregulated in HCC and may be associated with proliferation, migration, and EMT, potentially through interaction with the miR-2113/WDR66 regulatory axis (63). Regulation of FN1 splicing may be associated with FAK/AKT pathway activation and could be relevant to aggressive features of HCC (64). This indicates that the effect of SNRPA on HCC is associated with its role in the AKT-mTOR signaling pathway. Serum exosomal hnRNPH1 mRNA is elevated in HCC patients. It promotes the shift from active KHK-C to less active KHK-A isoforms. As a protein kinase, KHK-A drives HCC progression via Phosphoribosyl Pyrophosphate Synthetase 1(PRPS1) phosphorylation-induced nucleic acid synthesis and pentose phosphate pathway activation (65, 66). It is plausible that SNRPA may associate with AUUGCAC-containing regions in the pre-mRNA of these genes and thereby influence splicing-related processes, although this hypothesis will require further mechanistic validation (67, 68), and its upregulation by SNRPA may indicate their synergistic interaction.

While the present study provides an initial framework linking SNRPA overexpression to gene expression and alternative splicing changes in HCC cells, several limitations should be acknowledged. First, the study is based primarily on a single overexpression model in HepG2 cells, which limits both physiological relevance and causal inference. Given the current reliance on a single SNRPA overexpression model, loss-of-function experiments including siRNA-mediated SNRPA knockdown in HepG2 cells, as well as rescue assays, are planned for subsequent studies to verify whether SNRPA depletion can reverse the observed transcriptomic, splicing and proliferative alterations in HCC cells, which will help to establish a more rigorous causal relationship between SNRPA dysregulation and HCC progression. Additional loss-of-function, rescue, dose-response, and multi-model validation studies will be required to determine whether the observed effects are generalizable across HCC contexts. Second, although representative RASEs were validated, the functional consequences of individual splicing events remain unclear and will require isoform-specific perturbation and rescue experiments. Third, the initial splice-junction and AS-event catalogs included discovery-level candidates, and thus the relatively high proportion of novel events should be interpreted cautiously. Fourth, the iRIP-seq data provide region-level RNA-binding evidence rather than exact nucleotide-resolution maps; accordingly, overlap between binding targets and altered transcripts should not be taken as definitive proof of direct regulation. Finally, the translational relevance of these findings will require validation in patient-derived materials and assessment of their association with clinical outcome and therapeutic response in HCC.

## 5 Conclusion

In summary, our study shows that SNRPA overexpression in HepG2 cells is associated with widespread changes in gene expression and alternative splicing. Integrative RNA-seq and iRIP-seq analyses identified a subset of transcripts showing both SNRPA-binding evidence and altered expression or splicing patterns, supporting a potential role for SNRPA in RNA-related regulatory processes in HCC cells. However, additional loss-of-function, rescue, and higher-resolution RNA-binding studies will be required to establish direct mechanistic regulation. Subsequent *in vitro* functional assays (e.g., Transwell migration, colony formation) and *in vivo* xenograft models will be performed to validate the biological functions of key SNRPA target genes (CEMIP, EIF4A2, FN1) in HCC cell proliferation, migration and metastasis, and to further substantiate the regulatory role of SNRPA in HCC progression.

## Data Availability

The raw RNA-seq and iRIP-seq data generated in this study are available from the corresponding author upon reasonable request. Publicly available datasets analyzed in this study were obtained from the Gene Expression Omnibus (GEO) database under accession number GSE77509 (available at: https://www.ncbi.nlm.nih.gov/geo/query/acc.cgi?acc=GSE77509) and TCGA-LIHC cohort (available at: https://portal.gdc.cancer.gov/projects/TCGA-LIHC). All processed data, supplementary tables, and figures supporting the conclusions of this study are included within the article and its **Supplementary Materials**.

## Supplementary material

Supplementary Figure 1 Regulation of gene AS by SNRPA in HepG2 cells. **(A, B)** SNRPA regulation of the alternative splicing of *ECHDC2* and *PCBP2*.

Supplementary Figure 2 Candidate HCC-related splicing transcripts associated with SNRPA overexpression in HepG2 cells. **(A, B)** Representative alternative splicing events in PPP6R2 and GNAS associated with SNRPA overexpression.

Supplementary Figure 3 Candidate HCC-related splicing transcripts with SNRPA-binding evidence in HepG2 cells. **(A)** Representative alternative splicing event in HNRNPH1 associated with SNRPA overexpression. **(B)** Representative SNRPA-binding signal in HNRNPH1. Left: read density profile of the SNRPA-binding peak across the HNRNPH1 locus. Right: RIP-PCR/qRT-PCR validation of HNRNPH1 association with SNRPA.

Supplementary Figure 4 Candidate HCC-related splicing transcripts with SNRPA-binding evidence in HepG2 cells. **(A)** Representative alternative splicing event in FN1 associated with SNRPA overexpression. **(B)** Representative SNRPA-binding signal in FN1. Left: read density profile of the SNRPA-binding peak across the FN1 locus. Right: RIP-PCR/qRT-PCR validation of FN1 association with SNRPA.

## Glossary

A3SS: alternative 3’ splice site
A5SS: alternative 5’ splice site
ACP: Acid Phosphatase
AKR1C1: Aldo-Keto Reductase Family 1 Member C1
AS: alternative splicing
AXL: AXL Receptor Tyrosine Kinase
CCL20: C-C Motif Chemokine Ligand 20
CEMIP: Cell Migration Inducing Hyaluronidase 1
CLIP-seq: crosslinking-immunoprecipitation and High throughput sequencing
CYP4F2: Cytochrome P450 Family 4 Subfamily F Member 2
DERBPs: differentially expressed RBPs
DEGs: differentially expressed genes
ECHDC2: Enoyl-CoA Hydratase Domain Containing 2
EIF4A2: Eukaryotic Translation Initiation Factor 4A2
ES: exon skip
FDPS: Farnesyl Diphosphate Synthase
FN1: Fibronectin 1
GAPDH: Glyceraldehyde-3-Phosphate Dehydrogenase
GNAS: GNAS Complex Locus
GO: Gene Ontology
GTF2IP7: General Transcription Factor IIi Pseudogene 7
HCC: hepatocellular carcinoma
HepG2: human hepatocarcinoma cells
HISAT2: Hical Indexing for Spliced Alignment of Transcripts
HNRNPH1: Heterogeneous Nuclear Ribonucleoprotein H1
iRIP-seq: improved RNA Immunoprecipitation Sequencing
INHBB: Inhibin Subunit Beta B
LGALS3: Galectin 3
LIHC: liver hepatocellular carcinoma
LPS: Lipopolysaccharides
MALAT1: Metastasis Associated Lung Adenocarcinoma Transcript1
MFF: Mitochondrial Fission Factor
NC: normal cells
PCA: Principal component analysis
PCBP1: Poly(RC) Binding Protein 1
PCBP2: Poly(RC) Binding Protein 2
PLA2G2A: Phospholipase A2 Group IIA
PPP6R2: Protein Phosphatase 6 Regulatory Subunit 2
PRPS1: Phosphoribosyl Pyrophosphate Synthetase 1
RASEs: regulated alternative splicing events
RASGs: regulated alternative splicing genes
RBPs: RNA-binding proteins
RBM43: RNA-Binding Protein 43
RPS3: ribosomal protein S3
RNA-seq: RNA Sequencing
SIRT1: silent information regulator 1
SLC4A11: Solute Carrier Family 4 Member 11
SNRPA: Small Nuclear Ribonucleoprotein Polypeptide A
SNRPA-OE: SNRPA overexpression
SNRPD2: Small Nuclear Ribonucleoprotein D2 Polypeptide
SRA: Sequence Read Archive
STAT3: Signal Transducer And Activator Of Transcription 3
TCGA: The Cancer Genome Atlas Program
USP39: Ubiquitin Specific Peptidase 39
WDR66: WD repeat-containing protein 66.
