# Supplementary material for: Integrative RNA-seq and iRIP-seq analysis links SNRPA overexpression to transcriptomic and splicing alterations in hepatocellular carcinoma cells
Source: Front Oncol. 2026 May 1;16:1800728. doi: 10.3389/fonc.2026.1800728 (PMC13175842; doi:10.3389/fonc.2026.1800728)
Supplement: Supplementary Figure 1 — Regulation of gene AS by SNRPA in HepG2 cells. (A, B) SNRPA regulation of the alternative splicing of ECHDC2 and PCBP2. [file Supplementaryfile1.docx]

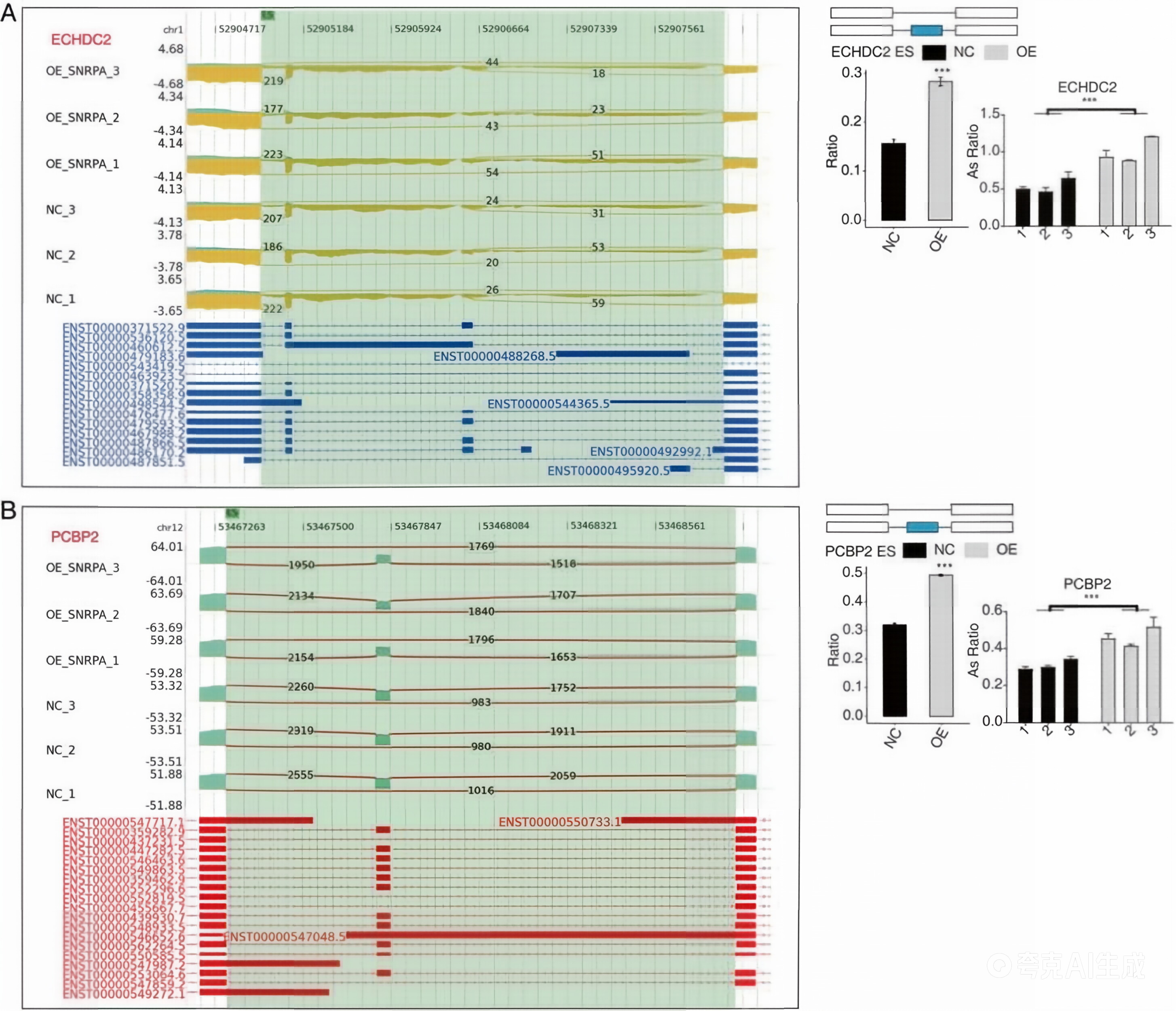


**Figure S1. Regulation of gene AS by SNRPA in HepG2 cells.**


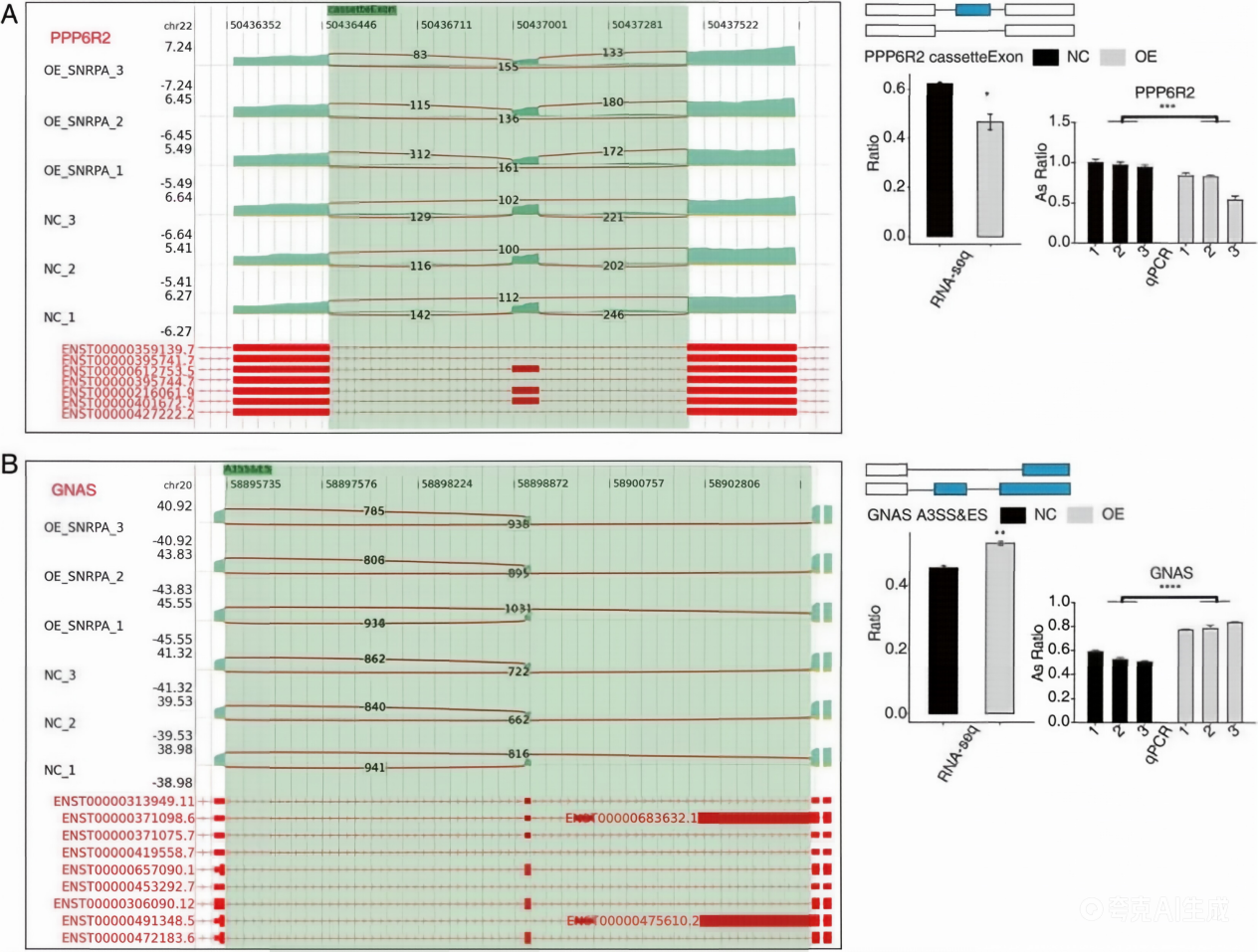


**Figure S2. Candidate HCC-related splicing transcripts associated with SNRPA overexpression in HepG2 cells.**

**
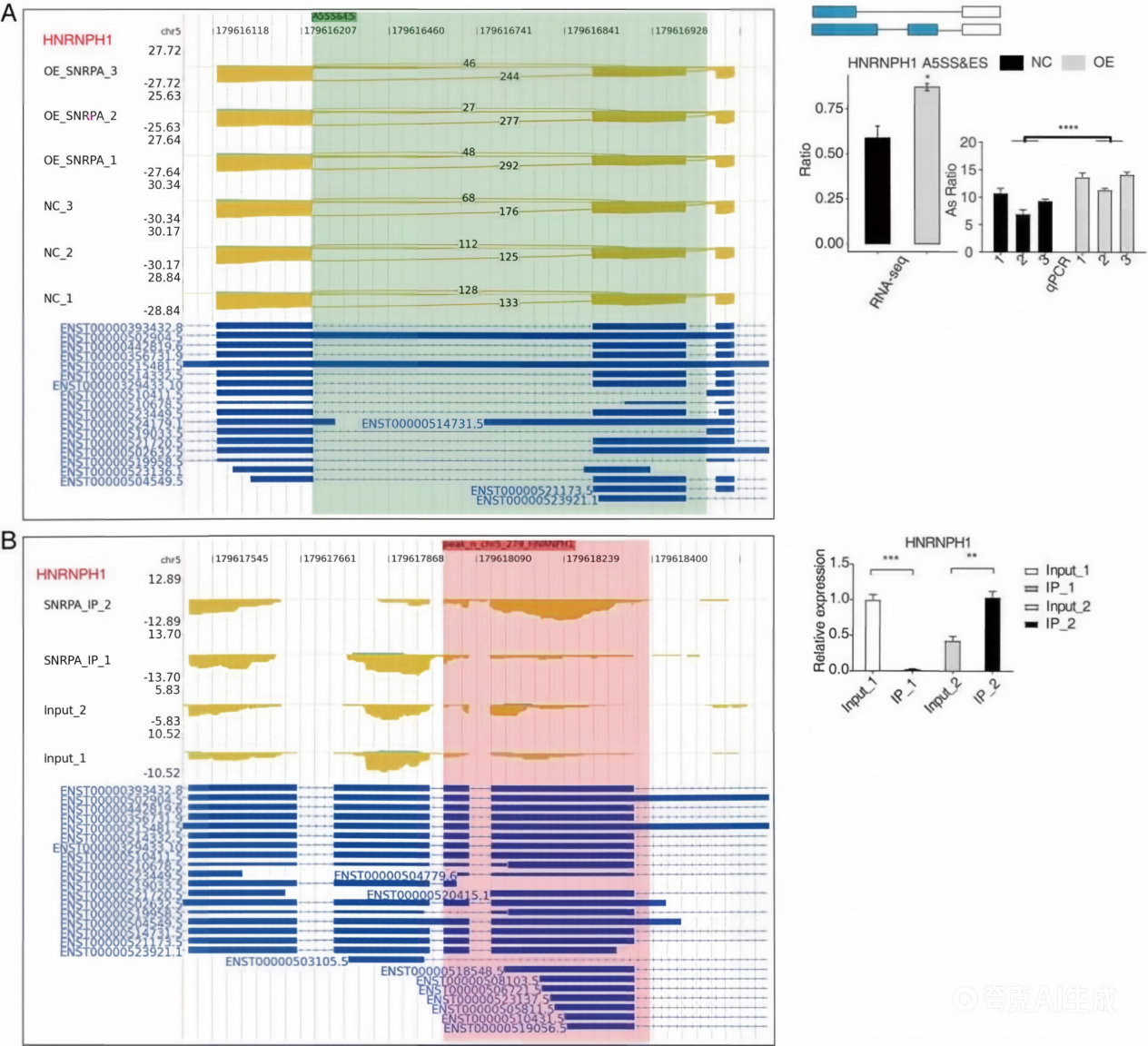
**

**Figure S3. Candidate HCC-related splicing transcripts with SNRPA-binding evidence in HepG2 cells.**

**
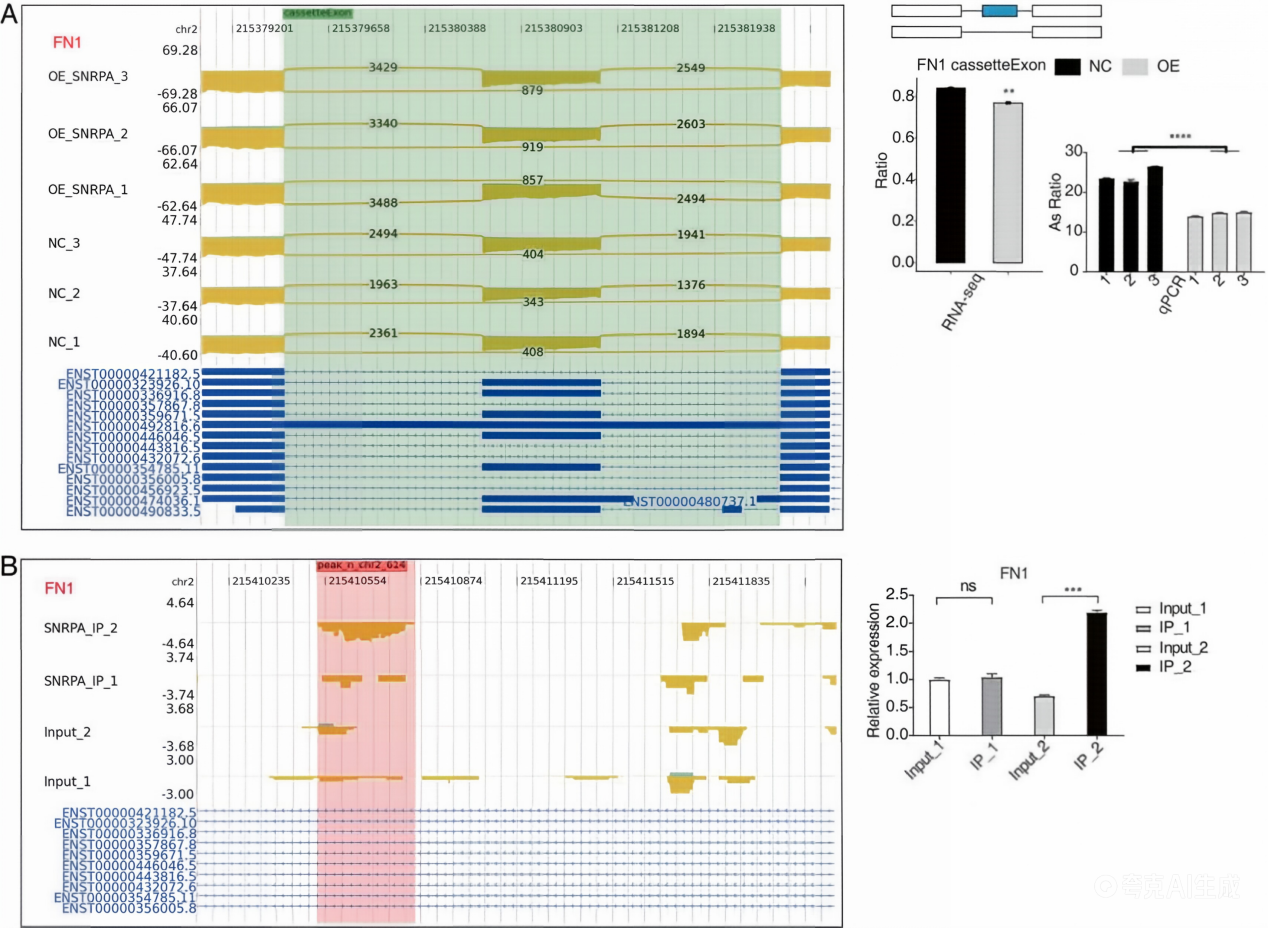
**


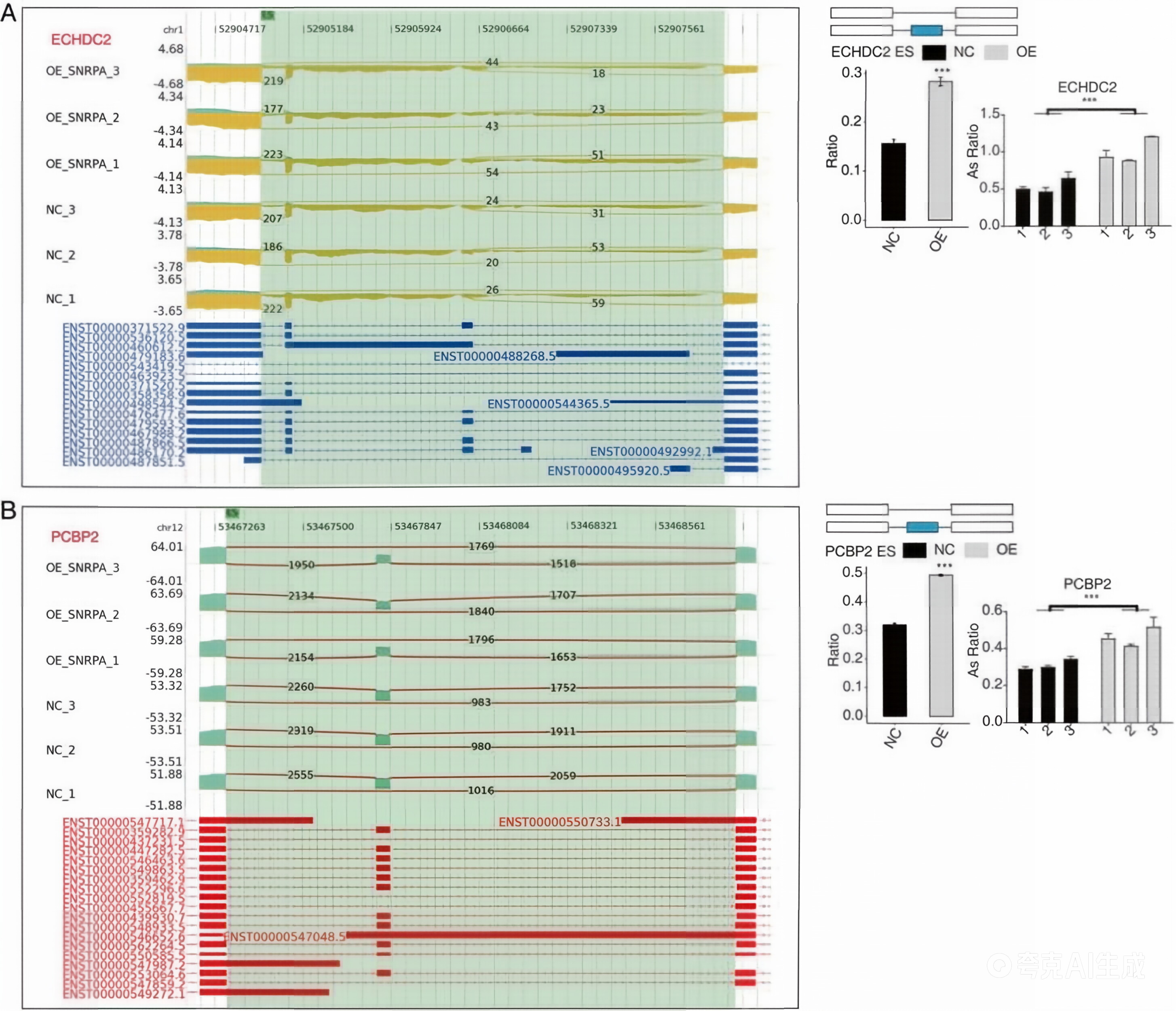


**Figure S4. Candidate HCC-related splicing transcripts with SNRPA-binding evidence in HepG2 cells.**
