# Supplementary material for: Integrative RNA-seq and iRIP-seq analysis links SNRPA overexpression to transcriptomic and splicing alterations in hepatocellular carcinoma cells
Source: Front Oncol. 2026 May 1;16:1800728. doi: 10.3389/fonc.2026.1800728 (PMC13175842; doi:10.3389/fonc.2026.1800728)
Supplement: Supplementary file 2 [file Table1.pdf]

Table.1 List of primers

Corresponding primers	Primer sequence （F）	Primer sequence （R）
hum-GAPDH	GGTCGGAGTCAACGGATTTG	GGAAGATGGTGATGGGATTTC
PLA2G2A	AGGGTATGAGAGAGGGAAAT	AGAAACAAGACGACCTACAA
INHBB	CGTTTCAGGGTATGAATGGA	TGAGGATTTAAGGATACACACC
CCL-20	GGAATGGAATTGGACATAGC	CAGTGAGGCACAAATTAGAT
AKR1C1	CAGTCCTTTGTGCCTTGG	TGCTCATTGTAGCTCTTGG
CEMIP	CATTCACCAAGAGCCAATATC	AGACAAGCATTTCAGAGGAG
CYP4F2	CACCATTTCAAAGTGTCCAA	GAGGAGCGACTGCTAATC
AXL	TCTAAAGGTCCACAGGTCTA	AGGAGGAAGCAATGATAGC
GTF2IP7	GCCAACACTTACCAGACT	CCTCAGCCAAGATGTCTC
LGALS3	AACCTGACCACTTCAAGG	GCACTGGTGAGGTCTATG
SLC4A11	GAAAGATCATCTTCATGTAGGG	CCAGAGGAAGATCCACTACT
FDPS - M / AS	TAGGAGTACCCGCCAACAAG	AGCAAAGGGCTCTGTTCCCAGAT
ECHDC２ - AS	TGAGTCCCTCCGATGTCATT	
ECHDC - M	GGGAAGGCTGCGATGTCATT	
ECHDC - M / AS		AGACCTGAAGGAGCGGGAAC
PCBP2-AS	GGATTCAGTGCAGGTTTGGAT	
PCBP2-M	AGGCTATTGGGCAGGTTTGGAT	
PCBP２ - M / AS		TCGTTTGGAATGGTGAGTTC
MFF - AS	TGGGCCCAGGGGGTGAGCAGG	
MFF - M	TTACACATTTGGTGAGCAGG	
MFF - M / AS		AGCGTTTGGCGGTGCTACTT
ACP１ - AS	TTGCCCGGCAGATTACCAAAG	
ACP１ - M	AGCAAGACAGATTACCAAAG	
ACP１ - M / AS		CAGATTGCTTTCATCCATAC
CPNE１ - M / AS	CAGATTGCTTTCATCCATAC	
CPNE１ - M		AGCTTTCCCCGATGGCCCACT
CPNE１ - AS		TCGCGCGGGGGATGGCCCACT
GNAS - M/AS	GTGCTGGAGAATCTGGTAAA	
GNAS - M		TTCGCCGCCCTCTCCATTAAA
GNAS - AS		CTTCTCACTGTCTCCATTAAA
PPP6R2 - M	TGTGGACCTGGCCTTCTCTGA	
PPP6R2 - AS	CCTTCAGCAGGCCTTCTCTGA	
PPP6R2 - M / AS		CTGGTCGGCAAACTCCTCAT
FN1-M	GCTGAACATTGGGTGGTGTC	GCAGTAACCACTATTCCTGCA
FN1-AS	GCTGAACATTGGGTGGTGTC	CAGTCCACAGCTATTCCTGCA
EIF4A2-M	CAACAAGTGTCTTTGGTTAT	TCTGCCAATTCTGTGAATAT
EIF4A2-AS	CAACAAGTGTCTTTGGTTAT	CTATCGACTCCTGTGAATAT
HNRNPH1-M	GCCACCGTAGCCGCCTCCGTA	TGGTGCTTATGCAAACCAGTC
HNRNPH1-AS	GCCACCGTAGCCGCCTCCGTA	ATGGGTTTGTCAAACCAGTC
primer binding site	AATTAACCCTCACTAAAGGG	GTCCTTATCATCGTCGTCTT
CMV	CGCAAATGGGCGGTAGGCGTG	
BGH		CGCAAATGGGCGGTAGGCGTG
CPNE１ - IP	ACCCGTTCAGGGGCTGCGGGAGT	ACCCGTTCAGGGGCTGCGGGAGT
EIF4A2-IP	TGCTAAGTGCTGTGTTGT	GTATGAGACCAAGCGTCC
SLC4A11-IP	GGAGTATCTGAGGGACACAT	GCCTGCATTCACATCCTG
CEMIP-IP	AACCCAGATTTCCCAGACT	GTAGCCGAGACAGTGTCA
PPP6R2-IP	TGCATAGCCGCTATTCTC	GTCACCATTGTGTTGTCAG
GTF2IP7-IP	TTCTCCTTGCCTCAACCT	GGCTCACGTCTGTAATCC
FN1-IP	CCACCTCCCAGGTTTAAG	CCTCTAATCCCAGCTACTC

Table 2. Obtain the high-quality clean reads											
SampleID	raw_total	clean_total	ratio_total	raw_base	clean_base	ratio_base	uniqtag	Q20	Q30	GC	DUP
NC_1	82552222	81713600	98.98%	12.383G	11.739G	94.80%	17907054<br>-43.77%	98.13	94.05	49%	71.31%
NC_2	69708614	69074362	99.09%	10.456G	9.881G	94.50%	17112534<br>-49.47%	98.15	94.04	48%	66.97%
NC_3	68380298	67772238	99.11%	10.257G	9.712G	94.68%	16544265<br>-48.74%	98.21	94.18	48%	67.01%
OE_SNRPA_1	76988922	76255042	99.05%	11.548G	10.915G	94.51%	18612978<br>-48.73%	98.14	94.04	48%	67.44%
OE_SNRPA_2	71497790	70854348	99.10%	10.725G	10.190G	95.02%	16945771<br>-47.76%	98.12	93.98	48%	67.41%
OE_SNRPA_3	71081342	70455044	99.12%	10.662G	10.107G	94.79%	16793715<br>-47.64%	98.21	94.2	48%	70.78%

Table3. Mapping of clean reads on the reference genome						
sample	NC_1	NC_2	NC_3	OE_SNRPA_1	OE_SNRPA_2	OE_SNRPA_3
Totalreads	80983582	68503168	67224454	75603636	70276376	69884536
Totalmapped	76538602	66112829	64821282	72786339	67819827	67522649
	94.51%	96.51%	96.43%	96.27%	96.50%	96.62%
TotalUniquely mapped	70069154	62719680	61307053	68999882	64309595	64098414
	91.55%	94.87%	94.58%	94.80%	94.82%	94.93%
TotalMultiplemapped	6469448	3393149	3514229	3786457	3510232	3424235
	8.45%	5.13%	5.42%	5.20%	5.18%	-5.07%
TotalPairs	40491791	34251584	33612227	37801818	35138188	34942268
TotalUniquelyConcordantPairs	33845502	30301108	29639183	33332541	31126646	31012071
	83.59%	88.47%	88.18%	88.18%	88.58%	88.75%
Splicereads	27408080	23887804	23675583	26052441	24344830	24311400
	39.12%	38.09%	38.62%	37.76%	37.86%	37.93%
Nonsplicereads	42661074	38831876	37631470	42947441	39964765	39787014
	60.88%	-61.91%	61.38%	62.24%	62.14%	62.07%

Table.4 ALL splicing Junction and AS events of samples from RNA-seq									
Sample	All_Junction	Novel_Junction	Known_Junction	knownAS(KAS)	AllAS	KAS%	novel AS(NAS)	AllAS	NAS%
NC_1	205429	52612	152817	28085	42926	65.43%	14841	42926	34.57%
NC_2	202623	51119	151504	27757	42962	64.61%	15205	42962	35.39%
NC_3	198268	48493	149775	26146	40286	64.90%	14140	40286	35.10%
OE_SNRPA_1	207716	53273	154443	28583	43874	65.15%	15291	43874	34.85%
OE_SNRPA_2	204810	51623	153187	27881	42435	65.70%	14554	42435	34.30%
OE_SNRPA_3	203417	50665	152752	27662	42559	65.00%	14897	42559	35.00%
Total	390716	196292	194424	49961	103706	48.18%	53745	103706	51.82%

Table.5 Classification of all RASE events between sample groups										
Sample	Type	3pMXE	5pMXE	A3SS	A3SS&ES	A5SS	A5SS&ES	ES	MXE	cassetteExon	Total
OE_SNRPA_vs_NC	Up	89	96	233	61	221	66	268	58	115	1207
OE_SNRPA_vs_NC	Down	73	74	257	27	243	38	112	50	235	1109
Total		162	170	490	88	464	104	380	108	350	2316

Table.6 High quality RNA - seq results

SampleID	raw total	clean_total	ratio_total	raw_base	clean_base	ratio_base	uniqtag	Q20	Q30	GC	DUP
Input_1	62325600	58879713	94.47%	9.349G	6.831G	73.06%	5593005 (19.38%)	97.5	92.65	66%	93.24%
Input_2	61948400	58393251	94.26%	9.292G	6.843G	73.65%	5542204 (19.35%)	97.48	92.65	66%	93.46%
SNRPA IP_1	52783600	45849564	86.86%	7.918G	4.824G	60.92%	5792380 (25.35%)	96.24	91.16	70%	92.25%
SNRPA IP 2	51740000	45988909	88.88%	7.761G	5.070G	65.32%	6217747 (27.18%)	96.17	90.92	71%	92.14%

Table.7 Comparison of effective sequencing data to a reference genome

Sample	Input_1	Input_2	SNRPA_IP_1	SNRPA_IP_2
Total reads	56139348	55579226	42446296	42619986
Total mapped	51030791(90.90%)	50295358(90.49%)	37081098(87.36%)	36588725(85.85%)
Total Uniquely mapped	11589400(22.71%)	11089959(22.05%)	6123215(16.51%)	7023805(19.20%)
Total Multiple mapped	39441391(77.29%)	39205399(77.95%)	30957883(83.49%)	29564920(80.80%)
Total Pairs	28069674	27789613	212223148	21309993
Total Uniquely Concordant Pairs	5391399(19.21%)	5146037(18.52%)	2790704(13.15%)	3193518(14.99%)
Splice reads	5828163(50.29%)	5479602(49.41%)	2477579(40.46%)	3255924(46.36%)
Nonsplice reads	5761237（49.71%）	5610357（50.59%）	3645636(59.54%)	3767881(53.64%)
rmdup reads	3327942(28.72%)	3257228（29.37%）	1606418（26.23%）	1939879（27.62%）
Sense reads	3174567（95.39%）	3109880（95.48%）	1470483（91.54%）	1805040（93.05%）
Antisense reads	153375（4.61%）	147348（4.52%）	135935（8.46%）	1348399（6.95%）

Table.8 The distribution of reads on the genome

Sample	5'UTR	3'UTR	CDS	Nc -exon	Introns	Intergenic	Antisense
Input-1	69423(2.09%)	165750(4.98%)	724564(21.77%)	256486（7.71%）	1152283（34.62%）	806057（24.22%）	153375(4.61%)
Input-2	68047(2.09%)	158345(4.86%)	688460(21.14%)	245982（7.55%）	1125255（34.55%）	823787（25.29%）	147348(4.52%)
SNRPA-IP-1	34414(2.14%)	48324(3.01%)	201147(12.52%)	103229（6.43%）	514437（32.02%）	568929(35.42%)	13593(8.46%)
SNRPA-IP-2	40916(2.11%)	58284(3.00%)	250922（12.93%）	126494（6.52%）	640432（33.01%）	687988(35.47%)	134839(6.95%)
